#  Does Regular Use of a Complementary Medicine of *Olea Europe *and *Ficus carica *Have Adverse Effects on Lipid Profile and Fasting Blood Glucose of Rheumatoid Arthritis (RA) Patients Under Treatment with DMARD Regimens Containing Methotrexate?

**Published:** 2016

**Authors:** Shahnaz Bahadori, Arman Ahmadzadeh, Mohammad Reza Shams Ardekani, Mohammad Kamalinejad, Mansoor Keshavarz, Jamshid Salamzadeh

**Affiliations:** a *Faculty of Traditional Medicine, Tehran University of Medical Sciences, Tehran, Iran.*; b *Department of Rheumatology, Loghman-e Hakim Hospital, Shahid Beheshti University of Medical Sciences, Tehran, Iran. *; c *Faculty of Pharmacy, Tehran University of Medical Sciences, Tehran, Iran.*; d *Department of Pharmacognosy, School of Pharmacy, Shahid Beheshti University of Medical Sciences, Tehran, Iran. *; e *Department of Physiology, School of Medicine, Tehran University of Medical Sciences, Tehran, Iran.*; f *Food Safety Research Center, Shahid Beheshti University of Medical Sciences, Tehran, Iran. *; g *Department of Clinical Pharmacy, School of Pharmacy, Shahid Beheshti University of Medical Sciences, Tehran, Iran.*

**Keywords:** Rheumatoid arthritis, Lipid profile, Fasting blood sugar, Olive, Fig

## Abstract

Rheumatoid arthritis (RA) patients are vulnerable to cardiovascular morbidity and mortality in which atherosclerosis plays a major role. In this study, the lipid profile and fasting blood sugar (FBS) of RA patients receiving a complementary medicine of olive and fig, as add-on therapy for routine disease-modifying antirheumatic drugs (DMARDs) regimen containing low dose methotrexate (MTX), were studied. A randomized controlled clinical trial was designed. Adult RA patients were randomly allocated in two groups receiving routine DMARDs regimen (control group) and routine DMARDs regimen plus the herbal supplementary formulation of olive oil, fig and olive fruits (intervention group). Patients were followed every 4 weeks for total study period of 16 weeks. In addition to demographic and medical history of the patients, the total cholesterol (TC), low-density lipoprotein cholesterol (LDL-C), high-density lipoprotein cholesterol (HDL-C), triglyceride (TG), the atherogenic index of plasma (AIP) defined as log(TG/HDL-C), and the fasting blood sugar (FBS) were determined and recorded. 56 patients (control = 27 and intervention = 29), with mean ± sd age of 50.9 ± 12.3 years completed the study. Average MTX dose received by intervention and control groups were 24.30 ± 18.39 and 17.61 ± 15.53 mg/week, respectively (p = 0.11). Repeated measures analysis of variance (ANOVA) revealed that differences between lipid profile indicators and FBS in the two study groups were not statistically significant (P>0.05). No additional substantial adverse reaction was seen in the study groups. Our findings are more reassuring for patients and their doctors to trust on the safety of the investigated complementary preparation to be used as add-on therapy to manage rheumatoid arthritis.

## Introduction

RA is characterized by acute and chronic inflammation in the synovium, which is associated with a proliferative and destructive process in joint tissues (1). Patients with rheumatoid arthritis (RA) are at increased risk of morbidity and mortality, leading to a reduced life expectancy in these patients (2-4). Evidence suggests that the increased morbidity and mortality rates in RA patients are largely associated with increased cardiovascular (CV) death that is mainly due to accelerated atherosclerosis (2-3, 5-6).

In addition, different studies have shown that effective treatment of RA patients with disease-modifying antirheumatic drugs (DMARDs) is associated with dyslipidemia. Dyslipidemia characterized with elevated plasma total cholesterol (TC) and low density lipoprotein cholesterol (LDL-C), as well as decreased high density lipoprotein cholesterol (HDL-C), is known as a major risk factor for cardiovascular diseases (CVD) in particular atherosclerotic coronary artery disease (CAD). Use of low dose methotrexate (MTX), the main agent recommended as mono, double and triple DMARD therapy regimens for RA (7), is associated with elevated expressions of atherogenic lipid profile (2). Westlake *et al*. in a review article in 2010, concluded that there is not enough evidence to determine the effect of MTX on traditional risk factors of CVD, particularly lipid profile, in patients with RA (8). Later in 2015, Vecchis *et al*. in a meta-analysis showed that there is a favorable profile of cardiovascular safety for MTX (9). They concluded since methotrexate does not interfere with blood lipids, platelet aggregation or insulin resistance, the protective association may originate from mechanisms other than those provided by antiplatelet drugs or statins. Other publications also confirmed a significant variation in lipid profile of RA patients after MTX therapy, which were mainly featured as increase in HDL-C and decrease in LDL- and VLDL-C (10, 11). 

In addition to lipid profile, it was shown that fasting serum glucose and serum insulin levels in early untreated rheumatoid arthritis patients are higher than those of the patients in the matched control group (12).This altered glucose metabolism can accelerate atherosclerosis in RA patients (8).On the other hand, MTX, used as a DMARD, can evidently increase glucose uptake and lipid oxidation in skeletal muscle (13). The mechanism by which MTX improves glucose homeostasis in patients with RA remains undetermined (14).

Olive (*Olea Europe*) and fig (*Ficus carica*) are amongst those herbal medicines that are frequently studied and documented to have beneficial anti-inflammatory, immunomodulatory, antimicrobial, anticancer, chemopreventive, analgesic and anti-oxidant effects. These biologic properties are mainly resulted from their oleic acid and phenolic components (15-31). It is documented that olive oil, in particular extra virgin olive oil, can improve lipid profile, through a decrease in total cholesterol (TC) and low-density lipoprotein cholesterol (LDL-C) and an increase in the ratio of HDL to TC. Unlike serum lipids, reports show that olive oil has no effects on glucose metabolism. In addition, olive oil, owing to its antioxidant polyphenol content, can increase antioxidant capacity in plasma, prevent oxidative damage or improve antioxidant enzyme function (32). These effects can lead to inhibition of the adverse inflammatory consequences in patients with RA.

Figs in both the fresh and dried forms contain considerable amounts of soluble fiber. Once figs are immersed in water, their viscosity increases and intestinal transit time may be extended, gastric emptying postponed as well as glucose absorption diminished. These effects have the potential to reduce postprandial blood glucose levels and decrease blood cholesterol (33). In a contradictory finding, reported by Peterson *et al*., it was revealed that figs-added diets could not change LDL-C, HDL-C and triglyceride concentrations compared to usual diet, whereas total cholesterol tended to increase significantly with fig consumption (33).

In our previous report, we showed that the supplementary medicine consisting of olive oil, olive (*Olea Europe)* and fig (*Ficus carica) *fruits could improve the Patient Global Assessment (PtGA) of the RA patients (34). Considering established experience of Iranian traditional medicine in herbal and nutritional therapy, we decided to design the current study with the aim of exploring the effects of a combination formulation of olive oil, olive and fig fruits on lipid profile and fasting blood glucose of patients with RA who are under treatment with DMARD regimens containing low dose (<25 mg/week) methotrexate.

## Material and Methods

A randomized controlled parallel group clinical trial of routine DMARDs regimen *vs. *routine DMARDs regimen plus an oral supplementary formulation of olive oil, olive and fig fruits (as add on therapy) was designed. Routine DMARDs regimen included methotrexate, hydroxychloroquine, azathioprine, sulfasalazine. The study was approved by the ethics committee of the Tehran University of Medical Sciences and was registered at the Iranian Registry of Clinical Trials with registration ID of IRCT2013122015876N1.

*Study population and sample size estimation*

Patients with definite diagnosis of RA referring to the in- and out-patient rheumatology departments of the Loghman-e Hakim University Hospital, Tehran, Iran, were randomly divided into two study groups receiving routine DMARDs regimen (control group) and routine DMARDs regimen plus the herbal supplementary formulation (an edible semisolid mixture) of fig and olive (intervention group). Sampling was carried out during September 2014 to August 2015. Estimated sample size for each group was 27 patients, with α = 0.05 and power = 80%. 

*Study herbal supplement*

The herbal supplement used in this trial was a combination of olive oil, olive fruit and fig fruit with proportional amounts of 2:5:1 w/w formulated as a semisolid mixture. An appropriate stabilizer, i.e. ascorbic acid, was also added to protect the product from oxidation. To ensure fresh formulation intake by the patients and for checking their compliance, study formulation was prepared and delivered to the patients in regular 10-day intervals. Patients were trained to take 15 grams (equal to 1 table-spoonful) of the mixture, t.d.s with meals and were asked not to change the usual dietary intake. They were also taught to keep the herbal medicine in a cool place away from heat and light. 

Olive and olive oil used in this study were prepared from the olive trees (*Olea europaea L*.) cultivated at Rudbar city located at the Gilan province in the North of Iran. Fig fruits were purchased as dried form, originated from common fig trees (*Ficus carica L*.) in Estahban city at the Fars province, Iran. Voucher specimens of the olive (Herbarium No: 1115) and fig fruits (Herbarium No: 8105) were preserved in the Herbarium of the School of Pharmacy, Shahid Beheshti University of Medical Sciences, Tehran, Iran.

*Study outcomes*

Primary outcome measure determined in this research, were plasma concentrations of the total cholesterol (TC), low-density lipoprotein cholesterol (LDL-C), high-density lipoprotein cholesterol (HDL-C), triglyceride (TG), the atherogenic index of plasma (AIP) defined as log(TG/HDL-C), and the fasting blood sugar (FBS). Patients were followed for 16 weeks. For each patient, an in-person follow up visit was arranged every 4 weeks. Therefore, there were 5 repeated measurements of the study variables for each individual patient; one at the time of enrollment (baseline) and 4 measurements during the 4-week intervals. All blood samples were obtained after 8-10 h of fasting.

In addition, demographic characteristics of the patients and their medical and medication history were recorded. 

*Inclusion and exclusion criteria*

All RA patients, male or female, over 18 years old with a DAS28_ESR score > 2.6 that were under treatment with DMARDs containing low-dose MTX entered the study. Exclusion criteria included use of any drug affecting lipid (*e.g. *selective & non-selective beta-blockers, thiazides and loop diuretics, alpha-agonist and antagonists, oral contraceptives) and glucose concentrations of plasma (except DMARDs), biologic agent therapy in the last 6 months, any addiction to psychotropic agents and opioids, patients with concurrent rheumatoid diseases and gout, patients with diabetes, regular consumption of olive and/or fig in the last 3 months, history of intraarticular corticosteroid in the last 3 months and pregnancy. In addition, exit criteria were any major change in the usual dietary intake, using any drug affecting plasma lipid and glucose levels, using other complementary and alternative medicine during the study period, any severe adverse reaction or intolerance to drug therapy including the herbal supplement, refusal for inclusion in the study and poor or noncompliant patients. Patients were asked to sign a written informed consent form before they enroll in trial.

*Statistical analysis*

Comparison of the demographic and baseline medical and medication history of the patients in two study groups were done using the Student’s t-test and Mann-Whitney U-test for quantitative data, and the Chi-square and Fisher’s exact tests for qualitative data. A repeated-measures analysis of variance (ANOVA) was applied to test any differences in repeated measurements of the total cholesterol (TC), low-density lipoprotein cholesterol (LDL-C), high-density lipoprotein cholesterol (HDL-C), triglyceride (TG), the atherogenic index of plasma (AIP) defined as log(TG/HDL-C) where TG and HDL-C presented as mmol/lit, and the fasting blood sugar (FBS) between control and intervention groups. p values < 0.05 were considered as significance level.

## Results

Overall, 72 patients entered into the study at the first step, of them 56 patients, 27 in the control group and 29 in the intervention group, completed the study. Mean ± sd of the MTX dose received by intervention and control groups were 24.30 ± 18.39 and 17.61 ± 15.53 mg/week, respectively (p = 0.11). Demographic data and baseline medical and medication history of the study groups and results of their comparison between two groups are presented at the Table1. As it is seen, no statistically significant difference existed in the demographic features, as well as medical and medication history of the patients in the control and intervention groups. Compliance of the patients in the intervention group was 93.88 ± 7.06 percent, ranging from 80% to 100%, confirming their appropriate adherence to the dosage regimen of the herbal supplement.

**Figure 1:**
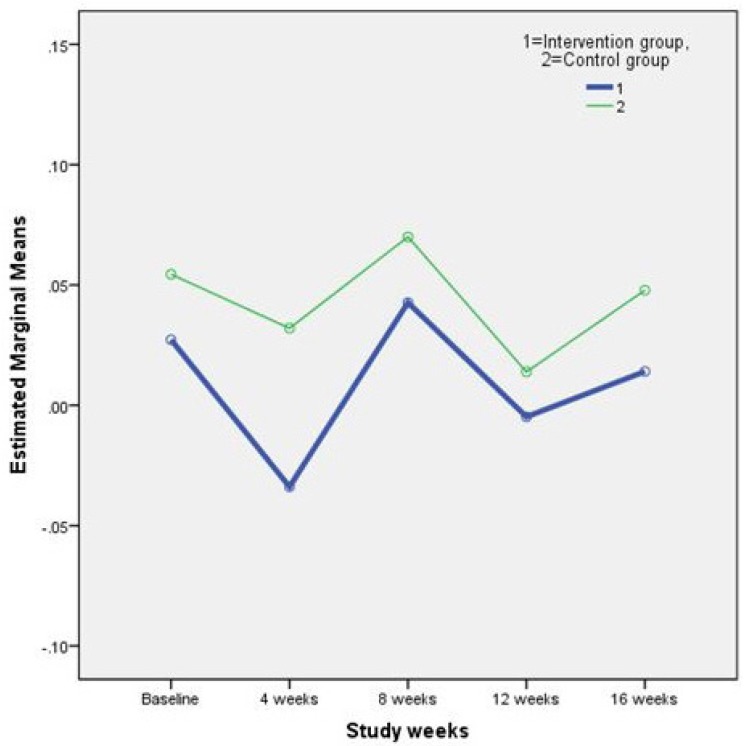
Changes in the mean Atherogenic Index of Plasma (AIP) during the study weeks.

Results of the repeated measures ANOVA on the plasmatic lipid profile and FBS are shown in Table 2. As it is perceived in the Table 2. differences between study outcomes in the two groups were not statistically significant. In other words, the supplement treatment used in this study, did not have any adverse effects on the concentration of plasma lipids and FBS. Figure 1. and 2. illustrate the changes in two main study outcomes of AIP and FBS, respectively.

**Figure 2:**
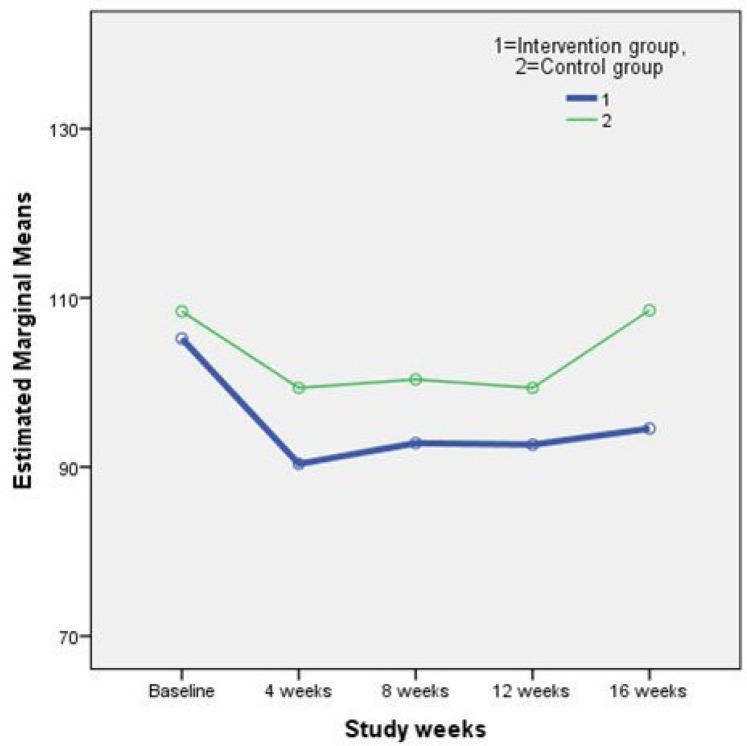
Changes in the mean fasting blood sugar (FBS) during the study weeks.

**Table 1:** Demographic data and baseline medical and medication history of the study groups

**Variable**	**Subgroup**	**Intervention group**	**Control group**	**p value**
Age (year)	N/A	50.38±12.25* range: (26-69)	51.48±12.49 range: (27-74)	0.74
Sex	Male	5 (17.24%)	3 (11.11%)	0.71
	Female	24 (82.76%)	24 (88.88%)	
RA history (years)	N/A	6.69±4.44 [6.00, 4.00-8.50]**	7.09±5.20 [6.00, 3.00-12.00]	0.98
Main referral reason	New case	3 (10.34%)	4 (14.81%)	0.81
	Flare-up	13 (44.83%)	10 (37.04%)	
	Follow up	13 (44.83%)	13 (48.15%)	
Concurrent rheumatoid disease	Yes	9 (31.03%)	9 (33.33%)	0.85
	No	20 (68.97%)	18 (66.67%)	
Comorbidity	Yes	8 (27.59%)	10 (37.04%)	0.45
	No	21 (72.41%)	17 (62.96%)	
MTX dose	N/A	24.30±18.39 [15.00, 10.00-50.00]	17.61±15.53 [10.00, 10.00-17.50]	0.11
Concurrent drug	Yes	8 (27.59%)	7 (25.93%)	0.89
	No	21 (72.41%)	20 (74.07)	
Total Cholesterol	N/A	183.56±31.05	188.50±37.10	0.51
LDL-C	N/A	95.40±24.91	108.33±28.93	0.62
HDL-C	N/A	54.00±23.83 [55.50, 40.75-61.00]	47.63±9.58 [47.00, 44.00-55.50]	0.29
TG	N/A	134.11±59.78 [109.00, 79.50-133.50]	136.58±102.58 [109.00, 76.75-157.25]	0.73
Atherogenic Index of Plasma	N/A	0.03±0.28	0.05±0.27	0.37
FBS	N/A	105.18±32.16 [92.00, 84.00-127.00]	108.41±32.49 [98.00, 85.00-125.00]	0.88
Menopause female patients	Yes	16 (66.67%)	15 (62.50%)	0.76
	No	8 (33.33%)	9 (37.50%)	

* Continuous data are presented as mean±standard deviation;

**[ Median, Interquartile range] for discrete or non-normally distributed variables; N/A: Not Applicable; RA: Rheumatoid Arthritis; MTX: Methotrexate; LDL-C: Low Density Lipoprotein Cholesterol; HDL-C: High Density Lipoprotein Cholesterol; TG: Triglyceride; FBS: Fasting Blood Sugar.

**Table 2:** Results of the repeated measures ANOVA on the lipid profile and FBS levels of the study groups

**Study** **outcome**	**Study** **group**	**Baseline**	**4 weeks**	**8 weeks**	**12 weeks**	**16 weeks**	**Multivariate** **p value**
TC (mg/dL)	Intervention	183.56±31.05	207.11±34.84	190.22±28.70	209.56±37.51	199.33±26.70	**0.25**
	Control	188.50±37.10	186.17±46.01	193.00±41.88	190.50±44.34	190.25±41.03	
LDL-C (mg/dL)	Intervention	95.40±24.91	122.60±31.39	105.76±21.23	113.56±17.66	115.40±13.76	**0.20**
	Control	108.33±28.93	101.82±28.45	105.96±30.02	112.03±36.25	98.87±30.62	
HDL-C (mg/dL)	Intervention	54.00±23.83	54.00±18.59	49.71±16.38	52.57±19.03	51.00±18.97	**0.25**
	Control	47.63±9.58	48.50±11.51	51.25±15.52	50.50±13.32	53.13±8.18	
TG (mg/dL)	Intervention	134.11±59.78	113.00±30.92	127.22±47.15	120.89±45.21	115.89±42.50	**0.21**
	Control	136.58±102.58	132.17±81.63	142.92±100.04	124.92±76.57	148.08±102.47	
AIP (mg/dL)	Intervention	0.03±0.28	-0.03±0.24	0.04±0.28	-0.005±0.30	0.01±0.30	**0.91**
	Control	0.05±0.27	0.03±0.28	0.07±0.33	0.01±0.28	0.05±0.26	
FBS (mg/dL)	Intervention	105.18±32.16	90.36±9.90	92.81±13.33	92.62±10.69	94.55±11.80	**0.84**
	Control	108.41±32.49	99.35±22.79	100.35±22.36	99.35±21.02	108.53±33.61	

ANOVA: Analysis of Variance; TC: Total Cholesterol; LDL-C: Low Density Lipoprotein Cholesterol; HDL-C: High Density Lipoprotein Cholesterol; TG: Triglyceride; AIP: atherogenic index of plasma defined as log(TG_(mmol/lit)_/HDL-C_(mmol/lit)_); FBS: Fasting Blood Sugar.

No other significant adverse reactions contributed to the investigational herbal supplement was seen in the study groups except one case with severe unclassified hiccup in the intervention group that leaded to his withdrawal from the study.

## Discussion

In the current study, possible adverse effects of a herbal supplement consisting of olive oil, olive and fig fruits, that its benefit on the global health of the RA patients had already been confirmed by our previous report (34), on plasma lipid profile and FBS of the same patients was investigated.

No significant variation in the concentrations of the cholesterol (TC), low-density lipoprotein cholesterol (LDL-C), high-density lipoprotein cholesterol (HDL-C), triglyceride (TG), the fasting blood sugar (FBS) as well as the atherogenic index of plasma (AIP), defined as log(TG/HDL-C), were obtained. In addition, study patients did not show an important adverse reaction contributed to the study complementary medicine. These confirm the safety of the investigated herbal preparation in the case of its effects on glucose and lipid metabolism, in patients with RA.

A cofounding factor that should be noted in the interpretation of our findings is that patients were under treatment with DMARDs regimens and this could have influenced the lipid profile of the patients in both groups. It is reported that patients in remission or with controlled disease show an increase in HDL-C levels and a reduction in the atherogenic index compared to patients with active disease (35). In addition, low dose MTX treatment has the potential to protect against dyslipidemia trough facilitation of cholesterol outflow (10).

In conclusion, considering the fact that RA patients are susceptible to atherosclerosis and cardiovascular death (2-3, 5-6), and that there are controversial reports about the influence of methotrexate on lipid profile of these patients (2, 8-11), our findings are more reassuring for patients and their doctors to trust on the safety of the investigated complementary preparation to be used as add-on therapy to manage rheumatoid arthritis. However, further investigations are encouraged to clarify the long-term effect of the study preparation. 
